# Study on The Reproductive Organs and Fertility of The Male Mice following Administration of Metronidazole 

**Published:** 2013-09-18

**Authors:** Mrinalini Kumari, Poonam Singh

**Affiliations:** Department of Zoology, MMV, Banaras Hindu University, Varanasi, India

**Keywords:** Epididymis, Metronidazole, Seminal Vesicle, Sperm, Testis

## Abstract

**Background:**

Metronidazole (MTZ) is commonly used as an antibacterial and antiprotozoal drug. Various doses of MTZ have been reported to inhibit spermatogenic activity and sperm indices.

**Materials and Methods:**

In this experimental study, dose-dependent effects of MTZ on the structural and functional integrity of the testis and accessory reproductive organs have been investigated. Adult male mice of Swiss strain were administered orally with MTZ at the doses of 250 mg/kgBW/day and 500 mg/kgBW/day for 28 consecutive days to study the changes in the testis, epididymis, seminal vesicle, sperm indices and fertility. Reversal effects of the drug were also studied on the same mice, 42 days after cessation of the treatment.

**Results:**

Therapeutic dose of MTZ (250 mg/kgBW/day) neither altered the weights of the testis, epididymis and seminal vesicle nor their histoarchitecture and sperm indices. The drug at the high dose (500 mg/kg BW/day) caused significant reductions in the weights of the testis and epididymis. Histoarchitecture of the testis and epididymis at the high dose revealed marked regressive changes while that of seminal vesicle remained unaffected. Significant reductions were noticed in the motility, viability and count of epididymal spermatozoa while the concentrations of epididymal sialic acid and seminal vesicular fructose remained unaltered after the treatment. No significant changes were noticed in the mating ability as well as in the level of serum testosterone in the treated mice. Fertility of the male mice treated with high dose of MTZ declined markedly leading to an increase in pre- and postimplantation loss while a significant decrease was noticed in the number of live blastocysts in females impregnated with such males. MTZinduced changes in the male reproductive organs and fertility were reinstated 42 days after cessation of the treatment.

**Conclusion:**

High dose of MTZ induced reversible deleterious effects on the male reproduction and fertility.

## Introduction

The increase in incidences of infertility in men due to frequent use of a number of therapeutic drugs has made efforts to study their untoward side effects on the male reproduction. Various drugs used for treating diseases are reported to cause male infertility (1, 2). Among them certain derivatives of nitroimidazole such as ornidazole, metronidazole, tinidazole and nimorazole are reported to impair the fertility potential by exerting adverse effects on the spermatogenesis and sperm parameters (3-6).

The first nitroimidazole to exert useful clinical activity is metronidazole, (MTZ; 1-[2-hydroxyethyl]2-methyl-5-nitroimidazole), a drug of first choice, recommended by the clinicians to be consumed at maximum for seven to ten days for the treatment of *Helicobacter pylori* infection, amoebiasis, giardiasis, trichomoniasis, bacterial vaginosis and several other anaerobic bacterial and parasitic infections. However, for the treatment of several complications like Chagas disease, Crohn’s disease, osteomyelitis, endocarditis, deep neck infection, joint infection and liver abscess, this drug is advised to be consumed for 4-8 weeks. Inspite of its long-term clinical use, untoward side effects of MTZ on the male fertility has been studied in laboratory rodents (3, 7, 8). Administration of various doses of MTZ (200 mg/kgBW/day and 400 mg/ kgBW/day) for 6 and 8 weeks causes suppressive effects on the spermatogenesis and fertility in the rats (3, 9, 10).

Quantitative studies have indicated marked alterations in the number of germ cells at stage I, V and XII following intraperitonial administration of 130 mg/kgBW/day of MTZ for seven days in mice (9) while the drug at the doses of 200 mg/kgBW/ day and 400 mg/kgBW/day for 60 days causes suppressive effect on spermatogenesis by altering the number of germ cells at stage VII of seminiferous tubule cycle in rats (10). Various doses of MTZ cause marked alterations in the count (3, 11, 12), motility (11, 12) and morphology of epididymal spermatozoa (3, 8) in laboratory rodents. Oda (13) reported dose-dependent decrease in the luminal content of epididymal spermatozoa in the MTZtreated rat. Decreased levels of gonadotropins and testosterone result in MTZ-induced suppressive effects on spermatogenesis (9-13).

From the foregoing it is clearly seen that MTZ at various doses impairs fertility in the males by inhibiting spermatogenic activity and sperm indices. However, a detailed study regarding the effects of therapeutic dose of MTZ for long duration, such as for 4-8 weeks on the male reproductive organs and fertility is still required. Therefore, the aim of the present study is to investigate the effects of the therapeutic and high doses of MTZ on the testis, epididymis, seminal vesicle and fertility as well as on the secretory activities of the latter two organs. For the safety evaluation of the potential effect of the drug on the male reproductive organs, a study with a dose higher than the therapeutic one may be considered in a non clinical trial. The study also deals with the withdrawal effects of high dose of MTZ, 42 days after cessation of the treatment. 

## Materials and Methods

### Animal selection

In this experimental study, fifty Swiss strain adult (12 weeks old) male mice weighing about 25-30 g were used for the present investigation. The animals were housed under standard laboratory conditions and maintained on pelleted diet and water ad libitum. Approval from the Animal Ethical Committee, Banaras Hindu University, Varanasi, India was obtained for the animal study plan (No. Dean/11-12/CAEC/263).

### Experimental design, drug and dosage

After recording the initial body weights, all the animals were divided into five groups of ten each and treated as follows:

Group I Untreated controls
Group II Vehicle-treated controls (distilled water)
Group III Administration of MTZ (250 mg/kgBW/ day) for 28 days
Group IV Administration of MTZ (500 mg/kgBW/ day) for 28 days
Group V Administration of MTZ (500 mg/kgBW/ day) for 28 days followed by sacrificing the animals 42 days after cessation of the treatment.

MTZ (CDH, India) was dissolved in double distilled water and administered orally. The human therapeutic dose of MTZ was selected and translated to mice (14). The doses 250 mg/ kgBW/day and 500 mg/kgBW/day of MTZ were administered to mice, equivalent to human therapeutic dose (20 mg/kgBW/day) and its higher dose (40 mg/kgBW/day), respectively. The procedure for the oral administration of the drug through gavage was based on the prior studies (8, 15).

### Animal sacrifice and collection of reproductive organs

After recording the final body weights the animals were sacrificed by cervical dislocation. Among ten animals from each group, five animals were used for the histological studies and sperm assessment while the other five were used for biochemical studies, fertility test and serum testosterone level. Blood was collected by cardiac puncture to measure the level of serum testosterone. The reproductive organs were dissected out, blotted free of blood and processed for the following studies:

#### Organs weight

Wet weights of the testis, epididymis and seminal vesicle were recorded to calculate the gonadosomatic index by using the following formula: Gonadosomatic Index (GSI)=(Gonad weight/total body weight) ×100.

#### Histological studies

Bouin’s fixed testis, epididymis and seminal vesicle were dehydrated and embedded in paraffin. Sections of 5 μm thickness were taken from the mid portion of each testis, all the three regions of epididymis and seminal vesicle, dehydrated in graded series of alcohol and stained with Periodic Acid Schiff reagent followed by counterstaining with Ehrlich’s Hematoxylin.

#### Quantitative study of the testis

Frequency of the stages was determined from one cross section of the testis of the five animals in each group. All the seminiferous tubules within a cross section of the testis were examined at ×40 and classified according to the stages of the cycle. The stages of the seminiferous tubules were classified according to the method of Hess and Franca (16). Due to severe degenerative changes in the seminiferous tubules, accurate identification of each stage was not possible; therefore, the tubules were grouped as stages I-IV, V-VI, VII-VIII, IX-X and XI- XII. The percentage frequency of all the grouped stages in one cross section of the testis in each of the five animals was calculated and analysed statistically. The relative number of each variety of germ cells at stage VII of the spermatogenic cycle (i.e. type-A spermatogonia (Asg), preleptotene spermatocytes (PLSc), pachytene spermatocytes (PSc) and stage 7 spermatids (7Sd)) was counted according to the method of Russell et al. (17).

#### Morphometric study of the seminiferous tubules

The diameter of the seminiferous tubules was measured using ocular micrometer at ×40 objective piece.

#### Biochemical studies

Concentrations of epididymal sialic acid and seminal vesicular fructose were estimated using the methods of Aminoff (18) and Linder and Mann (19) respectively.

#### Assessment of sperm parameters

Cauda epididymidis of five mice in each group was minced thoroughly in the physiological normal saline at 37˚C and used for the assessment of motility, viability and count according to the WHO Laboratory Manual (20). The sperm morphology was assessed by observing the smear prepared on a clean glass slide under microscope at ×40.

Evaluation of sperm abnormality was based on the criteria of Wyrobek and Bruce (21) and Zaneveld and Polakoski (22).

#### Serum testosterone assay

Serum testosterone was measured by ELISA, as described in the instructions provided in the kit (LDN, Germany).

### Mating ability and fertility

Each male was caged with two proestrus females overnight and according to presence of vaginal plug and implantation sites in females, the mating ability and fertility of the males were assessed respectively. The females were sacrificed by cervical dislocation on the fifteenth day of cohabitation with males. The ovaries were removed to count the number of corpus luteum. To determine the total number of implantation sites, the dissected out uteri were placed in 10% ammonium sulfide solution, which stained the hemosiderin pigment of resorbed implanted sites blue-black (23). The number of live implants, as well as pre- and post-implantation loss was recorded. Preimplantation loss was calculated using the following formula: 

Corpus luteum – [number of resorbed implants + number of live implants + number of dead implants] Postimplantation loss was equal to the total number of resorbed and dead implants.

### Statistical analysis

All the data were analyzed statistically by one way ANOVA followed by Newman-Keul’s test. 

Body weight and number of live implants as well as pre- and postimplantation loss were analyzed using Student’s t test. Values were considered significant at p<0.05.

## Results

### Body weight

No significant differences were found between the initial and the final body weights of the MTZtreated mice and the controls at therapeutic and high dose (Table 1). 

### Organs weight

Administration of MTZ at the therapeutic dose did not induce significant changes in the weights of the testis and epididymis while the drug at the high dose resulted in significant reductions in the weight of these organs as compared with the controls. Forty two days after cessation of the treatment, weight of the organs recovered to the control values. Administration of MTZ at any dose did not induce significant reduction in the weight of the seminal vesicle compared with that of controls (Table 1). 

**Table 1:** Effect of the oral administration of MTZ on body weight and weight of testis, epididymis and seminal vesicle (values are mean ± SE of five animals)


Groups	Body weight (g)		Weight of the reproductive organs (mg/100 gBW)		
	Initial BW	Final BW	Testis	Epididymis	Seminal vesicle

**I. Untreated control**	23.2 ± 1.35	28.0 ± 1.52	316.87 ± 20.6	131.36 ± 9.52	230.79 ± 22.41
**II. Vehicle-treated control**	23.8 ± 0.19	26.8 ± 0.58	310.40 ± 21.17	134.21 ± 6.15	230.84 ± 27.1
**III. MTZ (250 mg/kgBW/day)**	25.8 ± 0.48	28.4 ± 0.51	297.97 ± 9.09	122.54 ± 9.28	200.05 ± 20.11
**IV. MTZ (500 mg/kgBW/day)**	23.4 ± 1.32	27.8 ± 0.79	189.96 ± 4.95^a^	101.23 ± 5.3^a^	169.45 ± 14.53
V. MTZ (500 mg/kgBW/day)*	27.6 ± 0.51	33.2 ± 0.86	291.31 ± 9.94^b^	166.63 ± 8.29^b^	245.97 ± 25.57


*; Administration of MTZ for 28 days followed by sacrificing the animals 42 days after cessation of the treatment, a; As com- pared to Groups I and II: p< 0.05 and b; As compared to Group IV: p< 0.05.

### Histological studies

#### Testis

**Fig 1:**
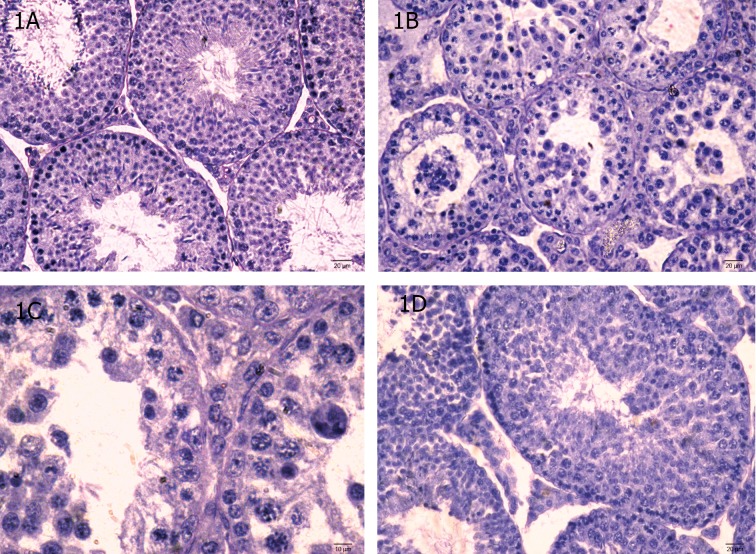
(A-D) Transverse section (T.S.) of the Testis of control (A) showing normal appearance of seminiferous tubules. (B-D) MTZ (500 mg/kgBW/day)-treated mouse for 28 days where (B) shows the shrinkage of the seminiferous tubules, depletion, disorganization, vacuolization and sloughing of the germ cells and appearance of multinucleated giant cells in the seminiferous tubules; (C) shows the giant cell (arrow); (D) shows the recovery in spermatogenesis in animals sacrificed 42 days after cessation of the treatment.

The testis of untreated and vehicle-treated controls (Fig 1A) showed normal histological features. MTZ at therapeutic dose induced mild regressive changes in the seminiferous tubules such as loosening of the germ cells only. However regressive changes in the seminiferous tubules appeared more pronounced in the testis of mice administered with high dose of MTZ. The changes included shrinkage of the seminiferous tubules, depletion, disorganization, intraepithelial vacuolization and sloughing of the germ cells (Fig 1B). Such changes were noticed in the seminiferous tubules in the testes of all mice, however, extent of regression varied from individual to individual. Giant cells containing round spermatids were also seen in some tubules (Fig 1C). Forty two days after cessation of the treatment, regressive histological changes noticed in the seminiferous tubules recovered completely in the testes of three animals out of five studied (Fig 1D).

#### Quantitative study of the testis

Quantitative analysis of the spermatogenic cycle revealed no alterations in all the stages of the seminiferous tubules in the testis of mice administered with therapeutic dose of MTZ as compared with the controls. In contrast, a significant decrease was observed in stages I-VIII after high dose of MTZ-treatment as compared with the controls (Table 2). Treatment with both doses of MTZ caused decrease in the germ cells in stage VII of the seminiferous tubules. However, reductions in the number of these cells in stage VII of the seminiferous tubules were significant in the testis of mice administered only with high dose of MTZ (Table 3). Forty two days after cessation of the treatment, number of cells at various stages of the seminiferous tubules (Table 2) as well as the different types of germ cells of stage VII recovered to that of control values (Table 3).

#### Morphometric study of the seminiferous tubules

Therapeutic dose of MTZ treatment did not induce any alteration in the diameter of the seminiferous tubules while a significant decrease in the same was noted in the testis of mice administered with high dose of the drug as compared with controls (Table 3). By 42 days after cessation of the treatment, tubular diameter recovered almost to the control value (Table 3).

**Table 2:** Effect of oral administration of MTZ on the percentage frequencies of stages of the spermatogenic cycle (values are mean ± SE of five animals)


Groups	Weight of the reproductive organs (mg/100 gBW)				
Stage I-IV	Stage V-VI	Stage VII-VIII	Stage IX-X	Stage XI-XII

**I. Untreated control**	28.05 ± 1.56	17.80 ± 3.29	27.37 ± 1.22	13.20 ± 1.73	15.53 ± 2.13
**II. Vehicle-treated control**	25.32 ± 1.56	23.18 ± 2.5	25.19 ± 3.87	11.99 ± 2.02	17.67 ± 4.05
**III. MTZ (250 mg/kgBW/day)**	25.64 ± 1.97	21.61 ± 0.9	25.98 ± 1.43	11.64 ± 1.09	15.09 ± 1.65
IV. MTZ (500 mg/kgBW/day)	06.85 ± 5.88 ^a^	12.91 ± 7.09 ^a^	05.99 ± 2.46 ^a^	36.93 ± 9.5 ^a^	37.27 ± 14.92
V. MTZ (500 mg/kgBW/day)*	23.64 ± 1.85 ^b^	23.05 ± 1.26 ^b^	24.43 ± 1.26 ^b^	10.69 ± 0.74 ^b^	18.14 ± 1.73


*; Administration of MTZ for 28 days followed by sacrificing the animals 42 days after cessation of the treatment, a; As com- pared to Group I and II: p<0.05 and b; As compared to Group IV: p< 0.05.

**Table 3:** Effect of oral administration of MTZ on the diameter and number of various types of germ cells of stage VII of the seminiferous tubules (values are mean ± SE of five animals)


Groups			Type A	Preleptotene	Pachytene	Round
	Diameter (μm)	spermatogonia	spermatocytes	spermatocytes	spermatids

**I. Untreated control**	215.55 ± 06.80	1.92 ± 0.19	51.32 ± 2.19	71.32 ± 4.22	175.00 ±14.25
**II. Vehicle-treated control**	223.28 ± 09.90	2.16 ± 0.31	49.44 ± 5.88	75.00 ± 8.08	174.34 ±18.90
**III. MTZ (250 mg/kgBW/day)**	218.96 ± 07.84	1.92 ± 0.30	40.36 ± 1.55	56.96 ± 3.36	147.92 ± 06.53
**IV. MTZ (500 mg/kgBW/day)**	179.71 ±11.20 ^a^	0.60 ± 0.60 ^a^	09.68 ± 9.60 ^a^	11.60 ± 11.6 ^a^	024.48 ± 24.40 ^a^
V. MTZ (500 mg/kgBW/day)*	198.72 ± 09.34	1.6 ± 0.28 ^b^	47.36 ± 5.13 b	79.04 ± 7.49 ^b^	159.36 ± 20.69 ^b^


*; Administration of MTZ for 28 days followed by sacrificing the animals 42 days after withdrawal of the treatment, a; As compared to Groups I and II: p< 0.05 and b; As compared to Group IV: p< 0.05.

#### Epididymis

**Fig 2:**
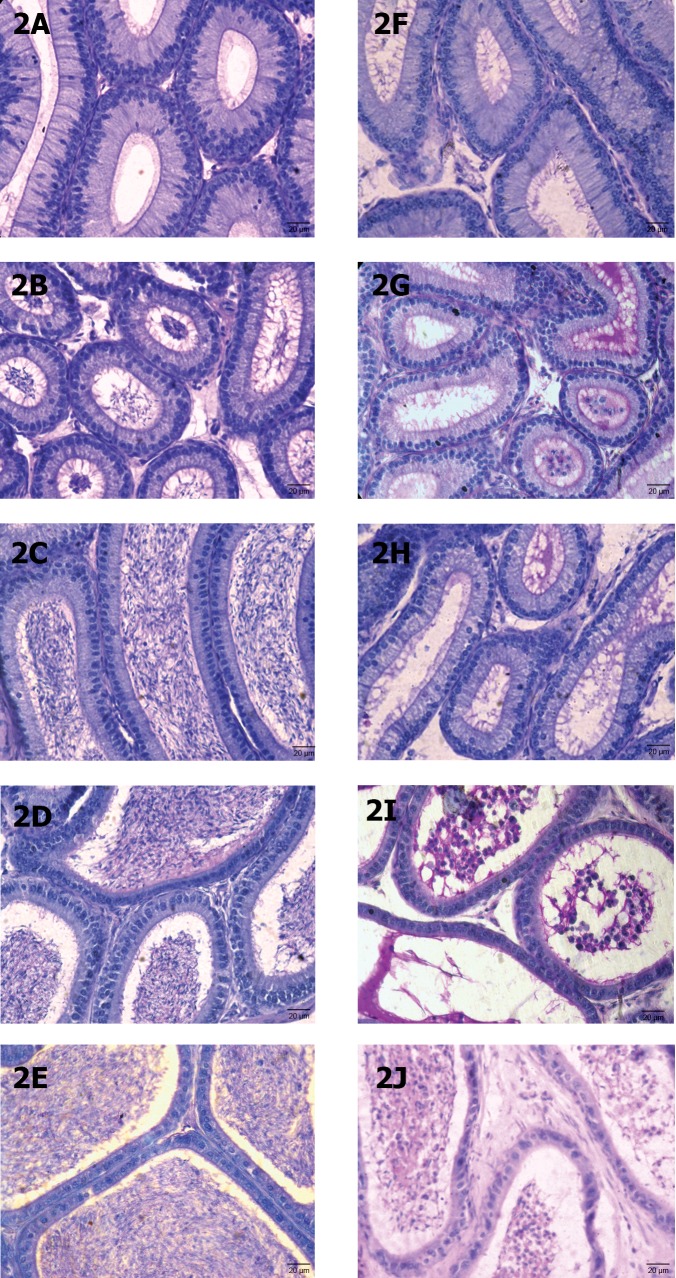
T.S. of various segments of the epididymis. (A-E) Segments of I-V of control to show normal histological features. (FJ) Segments of I-V of MTZ (500 mg/kgBW/day)-treated mouse for 28 days showing PAS-positive material, sperm debris and sloughed off germ cells in the lumina of segments II (Fig G), IV (Fig I) and V (Fig J).

The epididymis of the untreated and vehicletreated controls exhibited normal histological features. In the Swiss mice, five segments (I-V) were noticed in the epididymis. Segments I-III constituted the caput (Fig 2A-C); segment IV-corpus (Fig 2D) and segment V-cauda epididymides (Fig 2E). In mice treated with low dose of MTZ, these segments presented almost normal histology. High dose of MTZ-treatment caused no alteration in the first region of caput epididymidis (Fig 2F) while the same dose caused marked alterations in the lumina of second and third segments of caput (Fig 2G-2H) as well as in the corpus (Fig 2I) and cauda epididymides (Fig 2J), as indicated by presence of exfoliated germ cells and PAS-positive material with sperm debris. Increase in the fibromuscular stroma was also noticed in the cauda epididymides (Fig 2J). Forty two days after ceasing the treatment, spermatozoa reappeared in the epididymal lumen.

#### Seminal vesicle

**Fig 3:**
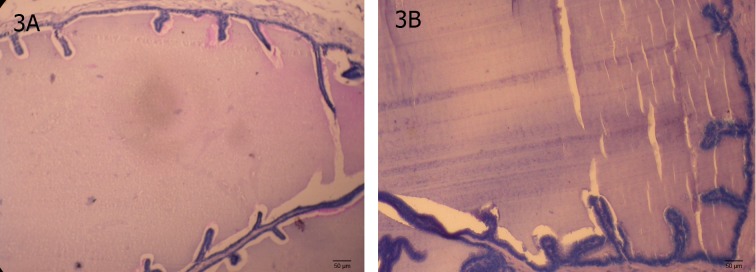
T.S. of the Seminal vesicles of control (A) to show normal histological features (B) MTZ (500 mg/kgBW/day)-treated mouse for 28 days showing unaltered histology.

Treatment with MTZ at any dose did not cause any alteration in the histoarchitecture of the seminal vesicle as compared with the control (Fig 3A and B).

#### Concentrations of sialic acid and fructose

Administration of MTZ at any dose did not induce significant alterations in the concentrations of sialic acid in the epididymis and fructose in the seminal vesicle (Table 4).

#### Epididymal sperm assessment

Therapeutic dose of MTZ did not cause significant reductions in the motility, viability and count of epididymal spermatozoa. By contrast these sperm indices declined significantly in mice administered with high dose of MTZ. Percentage of abnormal spermatozoa increased in MTZ-treated groups, though, the values were not significant. Withdrawal of the treatment, however, resulted in marked recovery in motility, viability and count of spermatozoa in the epididymis comparable to that of control (Table 5).

**Table 4:** Effect of the oral administration of MTZ on the concentrations of sialic acid in the epididymis and fructose in the seminal vesicle (values are mean ± SE of five animals)


Groups	Concentration of sialic acid (μmole/100 mg of tissue)	Concentration of fructose (μg/100 mg of tissue)

**I. Untreated control**	196.91 ± 46.08	264.53 ±15.4
**II. Vehicle-treated control**	195.71 ± 18.3	259.41 ±15.18
**III. MTZ (250 mg/kgBW/day)**	212.88 ± 41.26	237.75 ±13.78
**IV. MTZ (500 mg/kgBW/day)**	155.51 ± 22.55	235.09 ± 21.02
V. MTZ (500 mg/kgBW/day)*	214.21 ± 8.91	243.80 ± 15.73


*; Administration of MTZ for 28 days followed by sacrificing the animals 42 days after cessation of the treatment.

**Table 5:** Effect of the oral administration of MTZ on sperm motility, viability, morphology and count in the cauda epididymidis (values are mean ± SE of five animals)


Groups	Motility (%)	Viability (%)	Abnormal morphology (%)	Count (X 10^6^)

**I. Untreated control**	64.83 ± 2.69	63.25 ± 4	38.80 ± 5.47	13.98 ± 2.6
**II. Vehicle-treated control**	67.80 ± 6.82	64.95 ± 1.79	35.73 ± 5.43	13.66 ± 2.19
**III. MTZ (250 mg/kgBW/day)**	47.60 ± 4.76	53.67 ± 4.67	51.65 ± 5.59	09.84 ± 1.73
**IV. MTZ (500 mg/kgBW/day)**	28.23 ± 8.40 ^a^	23.41 ± 2.94 ^a^	62.40 ± 9.72	02.19 ± 0.34 ^a^
V. MTZ (500 mg/kgBW/day)*	65.78 ± 1.03 ^b^	64.76 ± 2.04 ^b^	42.73 ± 5.97	11.54 ± 1.4 ^b^


*; Administration of MTZ for 28 days followed by sacrificing the animals 42 days after cessation of the treatment, a; As com- pared to Groups I and II: p<0.05 and b; As compared to Group IV: p<0.05.

#### Serum testosterone level

No significant change was found in the level of serum testosterone caused either by therapeutic or high dose of MTZ as compared with the control (Table 6).

#### Mating ability and fertility

Mating ability of all the treated males remained almost unaffected comparable to that of the controls. Marked reduction was noted in the fertility of the males treated with high dose of MTZ; 67% of treated males became infertile after the treatment. Fertility of the virgin females impregnated with such males also declined by 75% (Table 7). In the remaining 25% fertile females, the number of live implants decreased significantly. An insignificant increase in the number of pre- and postimplantation loss was also noticed in such females (Table 8). Forty two days after cessation of the treatment, fertility of all the males recovered, however, only 34% of females showed recovery in their fertility when impregnated with such males (Table 7). The number of live implants as well as pre- and postimplantation loss was also recovered to some extent after withdrawal of the treatment (Table 8). 

**Table 6:** Effect of the oral administration of MTZ on the level of serum testosterone (values are mean ± SE of five animals)


Groups	Level of serum testosterone (ng/ml)

**II. Vehicle-treated control**	2.44 ± 0.2
**III. MTZ (250 mg/kgBW/day)**	2.42 ± 0.39
**IV. MTZ (500 mg/kgBW/day)**	2.14 ± 0.4
V. MTZ (500 mg/kgBW/day)*	2.32 ± 0.25


*; Administration of MTZ for 28 days followed by sacrificing the animals 42 days after cessa- tion of the treatment.

**Table 7:** Effect of the oral administration of MTZ on the mating ability and fertility of the males and the females (values are mean ± SE of five males and twelve females)


Groups	Males			Females		
	Tested	Mated	Fertile	Tested	Mated	Pregnant

**II. Vehicle-treated control**	6	6	6	12	12	12
**IV. MTZ (500 mg/kgBW/day)**	6	5	2	12	8	3
V. MTZ (500 mg/kgBW/day)*	6	6	6	12	8	7


*; Administration of MTZ for 28 days followed by sacrificing the animals 42 days after cessation of the treatment.

**Table 8:** Effect of the oral administration of MTZ on the number of live blastocysts and pre- and post-implantation loss (values are mean ± SE of twelve females)


Groups	Number of live blastocysts	Pre-implantation loss	Post-implantation loss

**II. Vehicle-treated control**	7.25	4.25	0.41
**IV. MTZ (500 mg/kgBW/day)**	2.75^a^	6.25	1.25
V. MTZ (500 mg/kgBW/day)*	4.5	5.16	0.16


*; Administration of MTZ for 28 days followed by sacrificing the animals 42 days after cessation of the treatment and a; As compared to Group II: p<0.05.

## Discussion

Oral administration of MTZ at therapeutic and high doses did not affect body weight of all the animals. However, significant reduction was noticed in the weight of the testis in the mice treated with high dose of MTZ. This is consistent with the findings reported in rats and mice (3, 7, 9-11, 13). Reduction in the testicular weight may be attributed to the depletion of the germ cell population (24). 

The histological study revealed that the therapeutic dose (250 mg/kgBW) of the drug did not cause marked alterations in the seminiferous tubules when administered for 28 days while the drug at high dose (500 mg/kg BW) for the same duration induced noticeable regressive changes in the seminiferous tubules resulting in the suppression of spermatogenic activity. McClain et al. have reported severe degeneration of the seminiferous tubules with appearance of giant cells in their lumina in the testis of rat exposed with MTZ at the dose of 400 mg/kgBW for 8 weeks. These authors have also reported partial recovery in spermatogenic activity three and a half months after cessation of the MTZ treatment (3). However, in the present study, almost complete recovery in spermatogenic activity was observed 42 days after cessation of the treatment in the testis of three animals out of five studied. The discrepancy between the present study and that of McClain et al. regarding recovery in spermatogenesis may be attributed to the exposure of MTZ for longer duration (8 weeks) (3). compared to the shorter duration (4 weeks) in our study. The present study further showed a decrease in the diameter of the seminiferous tubules in the testis only after high dose of MTZ treatment. This is consistent with the finding reported in Balb/c mice (15). Decrease in the diameter is attributed to cell death or exfoliation of the germ cells resulting in the shrinkage of the seminiferous tubules (25). Also, in the present study, sloughing or exfoliation of the germ cells has often been noticed in the seminiferous tubules in the testis of mice treated with high dose of the MTZ. The multinucleated giant cells observed in some of the regressed seminiferous tubules in the testis after treatment with high dose of MTZ is consistent with that observed in rat (13). These cells are considered to be an expression of germ cell degradation (26).

The spermatogenic inhibition as noticed in our study is reflected by alterations in the frequency of different stages as well as diminution of the germ cells at stage VII of the spermatogenic cycle. Earlier studies have shown that intraperitonial administration of 130 mg/ kgBW of MTZ for 7 days in CFW mice caused no alterations in the number of stages in the seminiferous tubules while the number of cells in stages I, V and XII was significantly increased (8). Quantitative study of Sohrabi and Mellati (10) has reported that oral administration of MTZ at the doses of 200 mg/kgBW and 400 mg/kgBW for 60 days caused significant reductions in the number of preleptotene spermatocytes and step 7 spermatid of stage VII of seminiferous tubule cycle in rats. In the present study, a significant decrease in stages I-VIII has been also observed with significant decrease in the number of type A spermatogonia, preleptotene spermatocytes, pachytene spermatocytes and round spermatids of stage VII seminiferous tubule of spermatogenic cycle in rat. The difference in the frequency of stages of the spermatogenic cycle is suggestive of alterations in the kinetics of spermatogenesis (27). The reductions in the germ cells of stage VII might be due to alterations in the hypothalamic-pituitary-gonadal axis feedback mechanism causing abnormal concentration of gonadotropins or testosterone (28) or due to the access of drug (Specify the drug) to the germ cells of seminiferous tubules through the blood-testis barrier (29) thereby resulting in spermatogenic arrest. In contrast to the findings of others (7, 9-11, 13), in our study, no significant reduction was noticed in the level of serum testosterone, therefore, suggesting the direct action of the drug on the spermatogenic activity. Tolnidamine, an indazole carboxylic acid, is also reported to cause direct effect on spermatogenesis without altering the androgen status in the Parkes mice (30). According to Edward et al. (31) the drugs belonging to the nitroimidazole group act through reduction of the nitro group in the cell which further oxidizes DNA thereby causing strand breaks and subsequently the cell death. In our study it might be possible that the drug at the high dose would have crossed the blood-testis barrier causing the germ cell death without inducing significant alteration in the level of serum testosterone thus indicating its direct effect on spermatogenesis. MTZ-induced oxidative stress in the testis has been reported by some other authors (11, 32). Our pilot studies (unpublished) have revealed significant alterations in the testicular antioxidant enzymes after MTZ-treatment. Therefore, oxidative stress-induced degeneration of germ cells may also be considered as a possible factor in spermatogenic inhibition.

A significant reduction in the weight of the epididymis in high dose of MTZ-treated mice is consistent with that reported by others (3, 8, 12, 13). MTZ-induced spermatogenic inhibition has probably resulted in reduction in the weight of the epididymis. Findings of McClain et al. (3) and Oda (13) have shown histological alterations in the epididymis exhibiting decrease in the luminal spermatozoa content after long-term administration of high dose of MTZ. Our histological observations have revealed absence of spermatozoa but indicated presence of sloughed off germ cells and PAS-positive material in the lumina of the corpus and cauda epididymides in the mice administered with MTZ at the dose of 500 mg/kgBW. Oda (13) has also reported appearance of sloughed off germ cells in the epididymal lumen in the MTZ-treated rat. Sialic acid is a true secretory product of the epididymis (33) and its secretion is testosterone-dependent (34). Therefore it appears that the unaltered level of the serum testosterone as noticed in the MTZ treated mice have not interfered in the secretory activity of the epididymis due to which the level of sialic acid remained unaffected.

MTZ is reported to inhibit the sperm motility at different doses in mice and rats (5, 7, 11). Likewise, in the present study significant low sperm motility was observed in a dose-dependent manner. Previous findings have suggested that the decrease in sperm motility by administration of ornidazole may be due to the inability of spermatozoa to obtain ATP through the glycolytic pathway (35) or due to the inhibition of energetic transferase or non-protein substance in the epididymis (6). Raji et al. (11) has reported reduced sperm motility due to alteration in the level of testicular SOD after MTZ administration. Therefore, based on our preliminary unpublished findings, we also cannot rule out the possibility of oxidative stress-induced decrease in sperm motility and viability.

Significant reduction in the sperm count noticed after high dose of MTZ treatment is consistent with the earlier findings (3, 11, 12). Decrease in the sperm count is the outcome of spermatogenic arrest following MTZ administration. In the present study an increased percentage of abnormal spermatozoa has been noticed in Swiss mice following oral administration of MTZ, though the values were not significant as compared with the controls. By contrast, findings of Mudry et al. (8) have reported a significant increase in the sperm cells abnormalities in CFW bred mice even with lower dose of MTZ (130 mg/kgBW) administered intraperitonially for 7 days. The discrepancy between our findings and that of Mudry et al. (8) may be because of the different responses exhibited by these two strains of mice and mode of administration of the drug.

In contrast to the reports of El-Nahas and ElAshmawy (7) and Sohrabi and Mellati (10) indicating a significant decrease in the weight of the seminal vesicle in MTZ- treated rodents, the present study revealed no significant reduction in the weight of the organ. Further, administration of MTZ at any dose did not alter the histology as well as the level of seminal vesicular fructose as compared with the controls. Since the structural and functional integrity of the accessory sex glands in the males are androgen-dependent (36), insignificant decrease in the level of serum testosterone as noticed in our study is probably not sufficient to alter the histoarchitecture as well as the fructose content of the seminal vesicle markedly.

MTZ administration at any dose did not affect the mating ability of the mice. However, marked reduction was noticed in the fertility of the males administered only with high dose of MTZ resulting in decrease in the fertility of females impregnated with such males. A consistent finding is reported in the rat (3). Decrease in the fertility of the treated males and increase in the pre- and postimplantation loss noticed in the females impregnated with such males, are possibly due to poor sperm quality which might have caused significant reduction in the number of live blastocysts. Recovery in fertility of the males and pre- and postimplantation loss in impregnated females 42 days after cessation of the treatment suggests that MTZ is not causing irreversible reproductive toxicity.

## Conclusion

High dose of MTZ induced rrelatively reversible deleterious effects on male reproduction and fertility, attributable to the direct action of MTZ on the spermatogenic activity rather than through serum testosterone depletion.
